# Minimum and Average Heart Rates in Pediatric Fontan Patients with Favorable Postoperative Outcomes: A Holter Monitoring Study

**DOI:** 10.1007/s00246-025-04106-x

**Published:** 2025-12-06

**Authors:** Peter Barrale, Alex R Sigman, Emily Pajazetovic, Emily Riley, Linder H Wendt, Aida Salameh, Jan Janoušek, Benjamin W Hale, Ian H Law

**Affiliations:** 1 Division of Pediatric Cardiology, Stead Family Children’s Hospital, University of Iowa Health Care, Iowa City, IA USA; 2 Roy J. Carver Department of Biomedical Engineering at the College of Engineering, University of Iowa, Iowa City, IA USA; 3 Department of Anatomy and Cell Biology, University of Iowa, Iowa City, IA USA; 4 Institute for Clinical and Translational Science, University of Iowa, Iowa City, IA USA; 5 Clinic for Pediatric Cardiology, Heart Centre, University of Leipzig, Leipzig, Germany; 6 Children’s Heart Center, 2nd Faculty of Medicine, Charles University in Prague and Motol University Hospital, Prague, Czech Republic

**Keywords:** Fontan, Heart rate, Bradycardia, Sinus node dysfunction, Holter, Ambulatory electrocardiogram, Single ventricle palliation, Minimum heart rate, Average heart rate

## Abstract

Fontan palliation is associated with an increased risk for arrhythmias and sinus node dysfunction. Although annual Holter monitoring is recommended for routine surveillance, there is no normative Holter data for this population, with only one study describing such data in healthy children. To describe Holter data for patients with Fontan physiology, compare this data to healthy children, and establish a normative data set for those undergoing routine ambulatory ECG screening. This retrospective, single-center study analyzed 642 Holter monitors from 133 Fontan patients aged 5–21 years, collected between 1970 and 2023. Patients with pacemakers, significant atrioventricular valve regurgitation, or severely depressed ventricular function were excluded. Demographic and clinical data were collected, and HR values were compared to a healthy control cohort. The Fontan cohort was predominantly male (69%), with right ventricular dominance (59%) and extracardiac Fontan palliation (80%). Compared to 502 healthy controls, Fontan patients had significantly higher minimum HRs in adolescents (14–16 years), lower average HRs in younger age groups (4–12 years), and universally lower maximum HRs. Within the Fontan group, males and those on digoxin had lower minimum HRs, while heterotaxy was associated with higher minimum and average HRs. This is the first study to establish normative Holter HR data in pediatric Fontan patients and compare it to healthy counterparts. These findings suggest Fontan patients do not have significantly lower minimum heart rates and provide a valuable reference for clinical evaluation, supporting the need for larger, multicenter studies.

## Introduction

Single ventricle (SV) physiology describes the group of congenital heart defects supported by one functioning ventricle with passive pulmonary blood flow [1, 2]. Single ventricle palliation was first published in 1971 by Dr. Francis Fontan, who palliated three patients with tricuspid atresia (TA) [3]. Following numerous surgical refinements and improvements in medical management, single ventricle palliation has become a suitable option for numerous complex congenital heart defects including hypoplastic left heart syndrome (HLHS), double outlet right ventricle (DORV), pulmonary atresia (PA), atrioventricular canal defects (AVC), transposition of the great arteries (TGA), double inlet left ventricle (DILV), Ebstein’s anomaly (EA), heterotaxy syndromes, and others otherwise not eligible for two ventricle repair [1, 2, 4 and 5]. Infants and children with these diagnoses require a stepwise surgical palliation to transition from parallel circulation to circulation in series via Norwood or hybrid palliation, hemi-Fontan or bidirectional Glenn, and Fontan procedures, culminating in passive systemic venous return to the lungs [1, 2 and 4].

The number of patients with SV or Fontan palliation continues to increase with an estimated 30-year survival of 85%, however congenital and surgical sequela increase the risk for heart and/or Fontan failure [1]. Arrhythmias are a common sequela for this population and may be worsened by atrioventricular (AV) valve regurgitation and ventricular dysfunction, which are independently associated with increased morbidity and mortality. The risk of specific arrhythmias varies with underlying anatomy and approach to SV palliation, but sinus node dysfunction and supraventricular tachycardia (SVT) remain common. While over time the rate of SVT and pacemakers has improved, sinus node dysfunction may be seen in up 44% of patients, independent of the underlying diagnosis or type of Fontan [1]. Patients with Fontan palliation had been reported to have bradycardia, likely secondary to sinus node dysfunction. Blaufox et al. reported that 27% of patients had a HR below the 5th percentile for age on resting electrocardiogram (ECG) [6].

The etiology of arrhythmia in this population is multifactorial, involving injury to the sinus node or its arterial supply, atrial suture lines, atrial dilatation, hypertrophy related to elevated atrial pressure, abnormal atrial tissue organization, programmed cell apoptosis, neonatal cyanosis, and complications of cardiopulmonary bypass [1]. Risk factors for late development of arrhythmias include age, atriopulmonary-type Fontan, perioperative arrhythmias, moderate or greater AV valve regurgitation, years post Fontan, male sex, right atrial isomerism, and loss of sinus rhythm during follow-up [1]. Arrhythmias are inherently more common in heterotaxy syndromes, such as left atrial and right atrial isomerism, as these conditions disrupt the development of the conduction system [7].

Ambulatory ECG devices, such as Holter monitors, have become routine screening tools for congenital heart disease (CHD), including Fontan palliation, by allowing extended ECG recording to better evaluate heart rates, rhythm, ectopy or additional beats, and arrhythmias [1]. While Holter monitors are frequently utilized, heart rate norms have traditionally been based on studies evaluating resting ECGs, which only provide a snapshot in time. At the time of this study, there was only one publication highlighting normal heart rate ranges in healthy children using 24-hour Holter monitors in 616 healthy pediatric patients with structurally normal hearts [8].

The current American Heart Association (AHA) guideline “Evaluation and Management of the Child and Adult with Fontan Circulation” published in 2018 recommends screening Holter monitors every one to three years to evaluate for sinus node dysfunction, increasing ectopy, and sustained or subclinical arrhythmia [1]. Despite this recommendation, there are no publications establishing the normal Holter values for this population. The goal of this study is to describe Holter data for patients with Fontan physiology, compare this data to healthy children, and establish a normative data set for those undergoing routine ambulatory ECG screening.

## Methods

This retrospective single-center study was approved by the Institutional Review Board (IRB) at the University of Iowa Health Care (IRB# 202302437). A total of 133 patients with Fontan palliation were included for a combined 642 total Holter monitors prior to 21 years of age. Event monitors were excluded due to intermittent recording. Numerous types of ambulatory ECGs were utilized, though 24- and 48-hour Philips monitors were most common.

Patients with successful Fontan palliation completed no earlier than 1971 were eligible for inclusion. All 24- or 48-hour heart monitor recordings completed prior to the patient’s twenty-first birthday in the electronic medical record were analyzed. Holter results were excluded if the patient had a 1.5 ventricle repair, Fontan take-down, or were post-heart transplant at the time of the Holter monitor. Patients with pacemakers programmed for atrial pacing at the time of the Holter were also excluded. If the most recent echocardiogram at the time of Holter (usually the done the same day) showed at least moderate-to-severe atrioventricular valve regurgitation (AVVR) and/or at least moderate-to-severe depressed function of the dominant ventricle, the associated Holter monitor was excluded. No patients in the cohort were documented as having undergone atrioventricular valve replacement surgery, which is less commonly performed at our center and typically reserved for palliative situations prior to Fontan completion or in cases of Fontan failure.

Patient demographic data including age, race, and sex were collected. Both age at Holter monitoring and time since Fontan completion were collected; however, age was used as the primary variable for analysis to enhance clinical applicability and reduce redundancy, given the strong correlation between these measures. Surgical variables included the patient’s single ventricle diagnosis, Fontan type, age at Fontan surgery, and if a fenestration was created. Fontan patients were categorized by dominant systemic ventricular morphology (right or left), as this was more consistently reported on echocardiography. Patients with borderline biventricular physiology were grouped by the dominant ventricle due to subjectivity in classification and low prevalence. Data on prior Glenn or hemi-Fontan procedures and sinus node artery involvement were not collected, limiting assessment of their impact on rhythm parameters; thus, this was not a study objective. Patient charts were reviewed to determine the presence of arrhythmias, pulmonary hypertension, dextrocardia, and anomalous pulmonary venous return prior to Fontan completion.

For each eligible Holter monitor, height and weight were determined from the closest measurements available in the patient chart. Data on all cardiac medications, including Beta blockers, digoxin, antihypertensives, diuretics, and pulmonary vasodilators were initially collected, however, to maintain clarity and focus on heart rate effects, only medications that would have a more direct effect on the heart rate (β-blockers and digoxin) were included in the analysis. If there was newly diagnosed arrhythmia or pulmonary hypertension, this was also documented. Heart rate data includes minimum HR, average HR, maximum HR, percentage of supraventricular and ventricular ectopy, presence of runs with four or more arrhythmic beats, significant arrhythmia during monitoring, and predominant heart rhythm. If significant ectopy or arrhythmia was found (more than 10% of total beats), the Holter was excluded. All Holter monitors were read by a pediatric cardiologist.

A recent echocardiogram—often performed the same day as Holter monitoring—was used to assess dominant ventricular function and atrioventricular valve regurgitation (AVVR). In patients with biventricular AV valve contribution, the valve with more significant regurgitation was used. Exercise testing was excluded due to heterogeneous modalities, inconsistent use prior to the 2019 guidelines, and our focus on establishing normative Holter benchmarks [1].

The ambulatory ECG results of the Fontan patients were compared with the results from the Salameh et al. study of healthy individuals without CHD. Categorical variables were summarized using counts and percentages, while continuous variables were summarized using medians and inter-quartile ranges. Fisher’s exact test was used to compare Holter results based on sex between the two studies, while the Wilcoxon rank sum test was used to compare all continuous variables of interest (age and minimum, maximum, and average heart rates) between both data sets. Within the Fontan subjects, univariate linear regression models were fit examining the predictive effect of sex, dominant ventricle, heterotaxy, Fontan type, and medications on heart rate measurements. For the multivariate models, an all-subsets regression procedure was used for each outcome in which all possible models among the candidate predictor variables (sex, dominant ventricle, age, heterotaxy, Fontan type) were tested and the model with the lowest Akaike Information Criterion (AIC) was selected. For all models, Beta coefficients and their corresponding 95% confidence intervals were presented along with p-values for each predictor. P-values less than 0.05 were considered statistically significant throughout the manuscript and R version 4.3.3 was used for all analyses.

## Results

### Comparing Fontan Patients to Controls

The demographic and heart rate characteristics of the Fontan population (Barrale) were compared to those of a healthy control pediatric population (Salameh et al.) using Fisher’s exact test and the Wilcoxon rank sum test. Table 1 describes the demographic data between groups. The sex distribution differed significantly between the two groups (*p* < 0.001). In the Fontan group, 31% were female (*n* = 200) and 69% were male (*n* = 442), whereas in the control group, 53% were female (*n* = 265) and 47% were male (*n* = 237). The median age of patients in the Fontan group was 12.2 years, which was significantly younger than the median age of 14.6 years in the control group (*p* < 0.001).

**Table 1:** Demographics and summary statistics comparing Fontan to healthy subjects

Characteristic	Fontan *n* = 6421	Control *n* = 5021	*p*-value^2^
Sex			< 0.001
Female	200 (31%)	265 (53%)	
Male	442 (69%)	237 (47%)	
Age (years)	12.2 (8.7, 15.4)	14.6 (10.8, 16.9)	< 0.001
Heart Rate Minimum (bpm)	48 (43, 58)	47 (43, 51)	< 0.001
Heart Rate Average (bpm)	81 (73, 90)	82 (74, 89)	0.7
Heart Rate Maximum (bpm)	154 (138, 171)	169 (151, 186)	< 0.001
Age			
[4,6)	60 (9.3%)	33 (6.6%)	
[6,8)	75 (12%)	34 (6.8%)	
[8,10)	71 (11%)	40 (8.0%)	
[10,12)	98 (15%)	60 (12%)	
[12,14)	112 (17%)	55 (11%)	
[14,16)	83 (13%)	103 (21%)	
[16,18)	72 (11%)	114 (23%)	
[18,20]	71 (11%)	63 (13%)	

Heart rate: values measured in beats per minute (bpm). Age: Brackets are inclusive, and parenthesis are exclusive, example [4–6) includes ages 4.0 to those less than 6 years old. ^1^n (%); Median (Q1, Q3) ^2^Fisher’s exact test; Wilcoxon rank sum test

Heart rate characteristics showed notable differences between the two groups (Table 1). The minimum heart rate was slightly higher in the Fontan group at 48 bpm compared to the control group (Fontan median 48 bpm, control median 47 bpm; *p* < 0.001), driven by the increased presence of high values in the Barrale study rather than a difference in medians. The average heart rate was similar between the two groups (Fontan median 81 bpm, control median 82 bpm; *p* = 0.7). However, the maximum heart rate was significantly lower in the Fontan group (Fontan median 154 bpm, control median 169 bpm; *p* < 0.001).

**Fig. 1:**
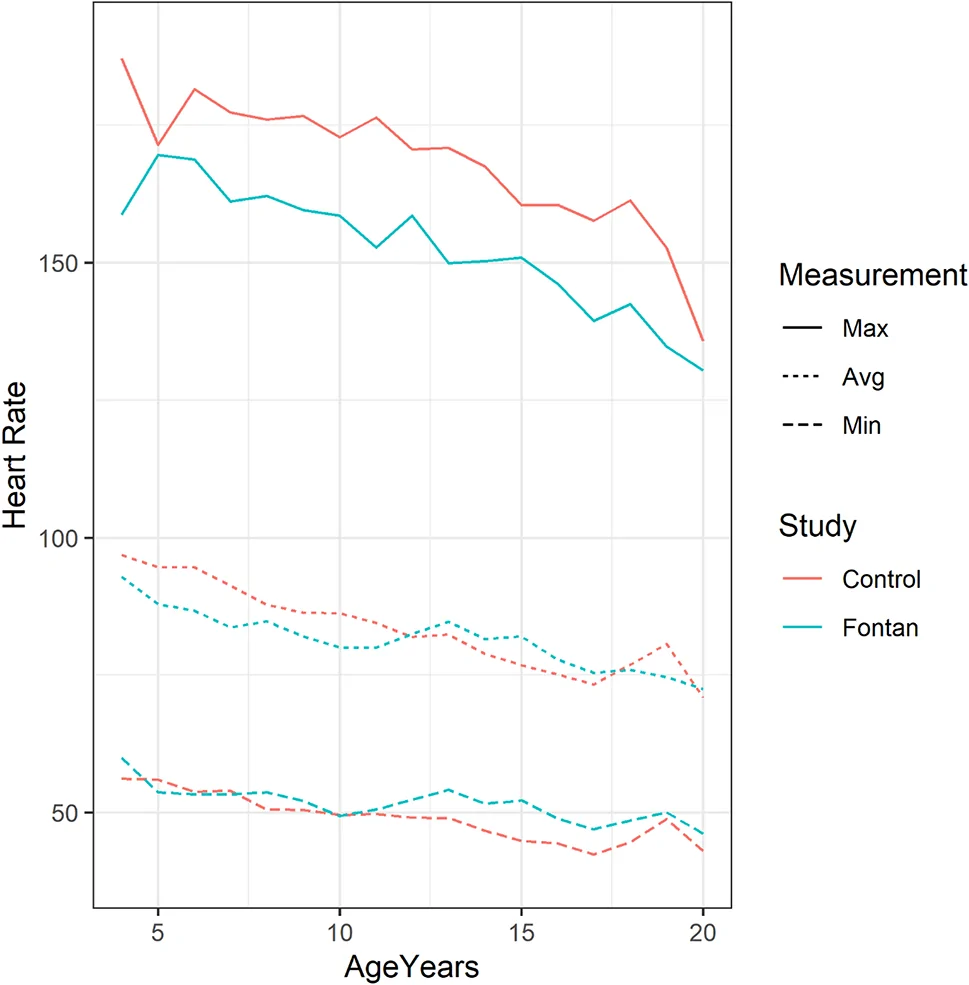
Comparison of Fontan Holter HR data to normal subjects. Trends highlight the minimum (dashed lines), average (dotted lines), and maximum HRs (solid lines) in Fontan subjects (Blue) compared to healthy control subjects (Red)

The age group distribution varied substantially between the two populations (Table 1), so the data was stratified and compared by 2-year age groups, noted in Table 2. Heart rate minimum, average, and maximum were compared. Fontan patients had similar heart rate minimums compared to their control counterparts. For minimum heart rate, the groups were similar except for significantly higher heart rates in the 12-14-year-olds (Beta 4.2 (95% CI 0.36–8.0); *p* = 0.032), 14–16-year-olds (Beta 6.1 (95% CI 3.4–8.8); *p* < 0.001) and 16–18-year-olds (Beta 4.6 (95% CI 2.0–7.3); *p* < 0.001). Average heart rates were significantly lower in the 4–6-year-olds (Beta − 6.2 (95% CI 11.0 – -0.89); *p* = 0.022), 6–8-year-olds (Beta − 7.4 (95% CI -13.0 – -1.9); *p* = 0.008),), and 10–12-year-olds (Beta − 5.2 (95% CI -8.8 – -1.6); *p* = 0.005). The only Fontan group that had significantly higher average heart rates were the 14–16-year-olds (Beta 3.9 (95% CI 0.73–7.1); *p* = 0.016). Fontan patients had significantly lower maximal heart rates in all age groups, with beta values ranging from − 13 to − 20 bpm (Fig. 1). Time since Fontan was recorded for all patients (*n* = 642), but was not used as a primary stratification variable due to its correlation with age at evaluation.

**Table 2:** Heart rate minimum, average, and maximum comparing fontan to healthy subjects

	Heart Rate Minimum			Heart Rate Average			Heart Rate Maximum		
Age	Beta	95% CI^1^	*p*-value	Beta	95% CI^1^	*p*-value	Beta	95% CI^1^	*p*-value
[4,6)	-0.41	-5.6, 4.8	0.9	-6.2	-11, -0.89	0.022	-13	-22, -3.5	0.008
[6,8)	-0.59	-5.7, 4.5	0.8	-7.4	-13, -1.9	0.008	-14	-23, -5.2	0.002
[8,10)	2.3	-2.1, 6.7	0.3	-3.8	-8.3, 0.69	0.10	-16	-23, -7.7	< 0.001
[10,12)	0.26	-2.7, 3.2	0.9	-5.2	-8.8, -1.6	0.005	-19	-26, -12	< 0.001
[12,14)	4.2	0.36, 8.0	0.032	1.5	-2.9, 5.9	0.5	-17	-24, -9.4	< 0.001
[14,16)	6.1	3.4, 8.8	< 0.001	3.9	0.73, 7.1	0.016	-13	-19, -7.3	< 0.001
[16,18)	4.6	2.0, 7.3	< 0.001	2.4	-0.44, 5.3	0.10	-16	-23, -9.2	< 0.001
[18,20]	3.1	-0.58, 6.8	0.10	-2.4	-6.2, 1.4	0.2	-20	-27, -13	< 0.001

Age: Brackets are inclusive, and parenthesis are exclusive, example [4–6) includes ages 4.0 to those less than 6 years old. ^*1*^CI = Confidence Interval. Beta represents difference in heart rate (BPM: beats per min) when comparing Fontan subjects. Positive beta represents a higher heart rate, and negative beta represents lower heart rate

### Analysis of Fontan Holter Data

The second portion of this study further analyzed the Fontan Holter data. Table 3 describes the demographic data. The cohort consisted of 200 females (31%) and 442 males (69%). The dominant ventricle was morphological right in 376 patients (59%) and morphological left in 266 patients (41%). Heterotaxy was present in 41 patients (6.4%), predominantly with right atrial isomerism, which accounted for 9 of 11 (82%) heterotaxy cases and 31 of 41 (76%) total Holter monitors. Regarding the type of Fontan procedure, 516 patients (80%) had an extracardiac Fontan, 117 patients (18%) had a lateral tunnel Fontan, 3 patients (0.5%) had an intra-extracardiac Fontan, 4 patients (0.6%) had a classical Fontan, and 2 patients (0.3%) had other/undisclosed types.

**Table 3:** Demographics and summary statistics of fontan holters

Characteristic	*N* = 642^*1*^
Sex	
Female	200 (31%)
Male	442 (69%)
Dominant Ventricle	
Left	266 (41%)
Right	376 (59%)
Heterotaxy	41 (6.4%)
Fontan Type	
Classic	4 (0.6%)
Extracardiac	516 (80%)
Intra/Extracardiac	3 (0.5%)
Lateral Tunnel	117 (18%)
Other	2 (0.3%)
Age (years)	12.2 (8.7, 15.4)
Time since Fontan (years)	8.7 (5.0, 12.1)
Medications	
No Beta Blocker or Digoxin	436 (68%)
Beta Blocker Only	27 (4.2%)
Digoxin Only	167 (26%)
Beta Blocker and Digoxin	12 (1.9%)
Age	
[4,6)	60 (9.3%)
[6,8)	75 (12%)
[8,10)	71 (11%)
[10,12)	98 (15%)
[12,14)	112 (17%)
[14,16)	83 (13%)
[16,18)	72 (11%)
[18,20].	71 (11%)

^*1*^n (%); Median (Q1, Q3). Age: Brackets are inclusive, and parenthesis are exclusive, example [4–6) includes ages 4.0 to those less than 6 years old

The median age of the patients was 12.2 years, and the median time since Fontan palliation was 8.7 years. Holter monitors were stratified and evaluated by age. All medications were collected, but only beta blockers and digoxin were evaluated as these have the most significant impact on heart rate. A total of 436 patients (68%) were not on beta blockers or digoxin, 27 patients (4.2%) were on beta blockers alone, 167 patients (26%) were on digoxin alone, and 12 patients (1.9%) were on both beta blockers and digoxin. Table 3 also shows the age distribution.

Univariate and multivariate analyses were performed to further evaluate the effect of collected variables on minimum, average, and maximum heart rate. Beta values describe the heart rate changes associated with each variable.

Minimum heart rate was significantly lower in males (*p* < 0.001, beta − 3.8 bpm) and those on digoxin (*p* = 0.045, beta − 2.4 bpm), and significantly higher in patients with heterotaxy (*p* = 0.005, beta + 5.8 bpm). Average heart rates were significantly lower in males (*p* = 0.009, beta − 3.0 bpm) and those on beta blockers alone (*p* = 0.003, beta − 7.9 bpm) or beta-blockers and digoxin (*p* = 0.028, beta − 8.6 bpm), but not in those on digoxin alone. Similar to heart rate minimum, heart rate average was significantly higher in patients with heterotaxy (*p* = 0.029, beta + 4.7 bpm). Heart rate maximum was significantly lower in patients with right dominant ventricles (*p* = 0.036, beta − 4.0 bpm), lateral tunnel Fontan (*p* < 0.001, beta − 13 bpm), those on beta blockers (*p* < 0.001, beta − 22 bpm), digoxin (*p* < 0.001, beta − 7.4 bpm), and the combination of beta blockers and digoxin (*p* < 0.001, beta − 13 bpm). Univariate analysis can be seen in Table 4. Multivariate analysis also showed males to have significantly lower heart rate minimum (*p* < 0.001, beta − 4.1 bpm) and average (*p* = 0.002, beta − 3.4 bpm). Patients with heterotaxy had significantly higher heart rate minimum (*p* < 0.001, beta + 3.4 bpm). Heart rate maximum was significantly lower in those with dominant right ventricles (*p* = 0.012, beta − 4.3 bpm) and lateral tunnel Fontan (*p* < 0.001, beta − 10 bpm).

**Table 4:** Univariate linear regression models predicting heart rate measures

	Heart Rate Minimum			Heart Rate Average			Heart Rate Maximum		
Characteristic	Beta	95% CI^1^	p-value	Beta	95% CI^1^	p-value	Beta	95% CI^1^	p-value
Male sex	-3.8	-5.9, -1.6	< 0.001	-3.0	-5.2, -0.74	0.009	2.9	-1.1, 6.8	0.2
Right Dominant Ventricle	0.10	-1.9, 2.1	> 0.9	-0.60	-2.7, 1.5	0.6	-4.0	-7.7, -0.25	0.036
Heterotaxy	5.8	1.8, 9.9	0.005	4.7	0.48, 9.0	0.029	1.2	-6.3, 8.7	0.8
Fontan Type									
Extracardiac	—	—	—	—	—	—	—	—	—
Lateral Tunnel	-1.5	-4.1, 1.1	0.3	-2.2	-4.9, 0.51	0.11	-13	-17, -8.1	< 0.001
Other	-1.5	-10, 7.1	0.7	-1.2	-10, 7.7	0.8	-10	-25, 5.1	0.2
Medications									
No Beta Blocker or Digoxin	—	—	—	—	—	—	—	—	—
Beta Blocker Only	-4.2	-9.2, 0.82	0.10	-7.9	-13, -2.7	0.003	-22	-30, -13	< 0.001
Digoxin Only	-2.4	-4.7, -0.06	0.045	-1.7	-4.1, 0.71	0.2	-7.4	-11, -3.4	< 0.001
Beta Blocker and Digoxin	-2.5	-9.9, 4.9	0.5	-8.6	-16, -0.94	0.028	-41	-54, -28	< 0.001

^*1*^CI = Confidence Interval. Beta represents difference in heart rate when comparing variables. Positive beta represents a higher heart rate, and negative beta represents lower heart rate

A comprehensive compilation of heart rate minimum, average, and maximum is summarized in Table 5, which is stratified by age and sex. For each age group and heart rate variable, the median, first and third quartiles, and range are provided.

**Table 5:** Heart rate values in patients with Fontan palliation

	Minimum HR		Average HR		Maximal HR	
Age	Female *N* = 200^1^	Male *N* = 442^1^	Female *N* = 200^2^	Male *N* = 442^2^	Female *N* = 200^3^	Male *N* = 442^3^
[4,6)	58 (49, 68) Range: 36–82	50 (44, 59) Range: 38–88	94 (91, 102) Range: 68–110	88 (80, 94) Range: 59–129	161 (158, 174) Range: 117–197	171 (156, 184) Range: 94–200
[6,8)	49 (44, 57) Range: 35–89	48 (45, 57) Range: 30–90	87 (76, 94) Range: 61–112	86 (70, 95) Range: 58–116	167 (146, 176) Range: 101–211	169 (148, 182) Range: 113–207
[8,10)	52 (49, 69) Range: 34–85	50 (42, 56) Range: 31–85	87 (81, 95) Range: 57–110	83 (71, 90) Range: 61–110	154 (140, 171) Range: 117–182	165 (150, 177) Range: 100–200
[10,12)	45 (42, 58) Range: 37–74	49 (44, 55) Range: 31–79	77 (70, 89) Range: 56–101	78 (73, 89) Range: 59–109	154 (140, 167) Range: 118–185	160 (143, 174) Range: 87–197
[12,14)	55 (47, 75) Range: 44–91	48 (43, 58) Range: 32–79	89 (79, 101) Range: 62–114	81 (73, 92) Range: 18–135	156 (136, 167) Range: 115–185	156 (143, 171) Range: 54–207
[14,16)	50 (46, 65) Range: 41–77	47 (42, 58) Range: 34–98	86 (79, 90) Range: 56–102	81 (74, 88) Range: 47–115	148 (138, 157) Range: 109–190	150 (135, 167) Range: 112–194
[16,18)	48 (42, 59) Range: 36–87	45 (37, 55) Range: 30–73	79 (75, 86) Range: 60–111	77 (68, 83) Range: 55–93	146 (132, 166) Range: 113–209	138 (128, 148) Range: 99–188
[18,20]	46 (42, 56) Range: 33–89	43 (36, 55) Range: 31–71	75 (66, 84) Range: 61–110	73 (65, 80) Range: 58–88	137 (124, 146) Range: 106–189	135 (115, 145) Range: 94–194

Age: Brackets are inclusive, and parenthesis are exclusive, example [4–6) includes ages 4.0 to those less than 6 years old. ^1^Min: Median (Q1, Q3), Range, ^2^Avg: Median (Q1, Q3), Range, ^3^Max: Median (Q1, Q3), Range

## Discussion

Patients with Fontan physiology remain a significant focus in pediatric cardiology. Advances in medical care have extended longevity. However, the increase in longevity has resulted in numerous sequelae due to physiological differences in their heart structure and palliative surgery. Arrhythmias remain a common sequela for Fontan patients, prompting the AHA recommendation for routine ambulatory ECG, as those with arrhythmias have poorer outcomes [1].

There is currently only one publication describing normal Holter values in healthy children and no publications that provide normal Holter values for the Fontan population. The goal of this study is to describe Holter data for individuals with Fontan physiology, compare Fontan Holter data to that of healthy children, and establish a normative data set for Fontan patients undergoing routine Holter monitoring.

When compared to normative Holter data in healthy children, patients with Fontan palliation did not have significantly lower heart rate minimums; in fact, those aged 12–18 years had significantly higher minimum heart rates. Younger Fontan patients (under 12 years) had lower average heart rates, with statistical significance for average heart rates in Fontan patients aged 4–6, 6–8, and 10–12 years. However, adolescents had higher average heart rates, with significance in the14-16-year-old group. Heart rate maximum was not further assessed due to the inconsistency and uncertainty of day-to-day activities, which is a common issue in ambulatory monitoring.

While previous ECG studies suggest that Fontan patients have lower resting heart rates, Fontan patients did not have statistically significantly lower heart rate minimums on Holter [6]. Average heart rates were lower in 4–8 and 10-12-year-olds; however, this may be difficult to interpret as heart rate maximums were significantly lower in all age groups, which will have a substantial impact on average heart rates.

Upon further review of the Fontan dataset, minimum heart rate was significantly lower in males and in those taking digoxin compared to other Fontan patients—findings consistent with healthy pediatric populations and attributable to digoxin’s parasympathomimetic effects [9, 10]. Average heart rates were also lower in males and in those on beta blockers, with or without digoxin. These sex-based differences in heart rate align with prior studies, though formal significance testing across all age groups was not performed. Interestingly, single-ventricle patients with Heterotaxy syndrome had significantly higher minimum and average heart rates—an unexpected finding given the known association with sinus node dysfunction in this population [7]. Further distinction between right and left atrial isomerism may yield additional insights, but subgroup sizes were too small for meaningful statistical comparison. Future studies with larger cohorts should explore rhythm differences across Heterotaxy subtypes. The potential impact of the Glenn procedure on rhythm was considered, particularly regarding sinus node artery injury, but procedural details were inconsistently documented; thus, this was not a study objective.

### Strengths

This study is the first to describe baseline heart rate Holter data in patients with Fontan palliation. Given the AHA recommendation for routine Holter monitoring in the Fontan population, this descriptive study allows single ventricle (SV) providers to compare their patients’ heart rates to a similar patient population, which is pertinent in both the inpatient (i.e. telemetry) and outpatient setting. The extended 24-to-48-hour monitoring period provides a more comprehensive understanding of heart rate ranges, rather than relying solely on baseline resting ECGs. Developing normative data also aids in comparing heart rates in patients with Fontan palliation to their healthy peers with structurally normal hearts. While numerous studies suggest that Fontan patients have lower minimum heart rates, this study suggests further clinical evaluation may be required if heart rates are significantly lower, given the lack of significant differences when compared to healthy counterparts.

### Weaknesses

An inherent limitation of this study is its retrospective nature. This study relies on previously recorded data, which may be subject to documentation errors, inconsistencies, or incompleteness. Not all subjects received yearly Holter monitors as clinically recommended, resulting in gaps in our data sets. Additionally, not all Holter monitors were conducted at our institution, leading to incomplete transfer of records and reporting, which required exclusion. The study also included sequential Holter monitors on the same patients, biasing heart rate data results toward patients who underwent multiple Holter monitors.

Our analysis included multiple observations per subject to increase representation across age groups in a small population, as limiting to one data point would have substantially reduced the sample size. A longitudinal approach was not feasible due to inconsistent Holter availability across patients, which we acknowledge as a limitation.

Conducting a retrospective study prevented control over factors like patient compliance, monitoring duration, and the quality of journaling and patient-recorded details. A prospective study would enable standardized data collection, ensuring higher accuracy and completeness. Activity levels were not documented, potentially biasing maximum heart rate data due to patient inactivity while wearing a Holter, especially in a population that is less active [11]. This limitation also exists in the Salameh study. Additionally, we cannot quantify the number of hours patients were awake or sleeping. Recording artifacts are inherent to all ambulatory monitoring, introducing variability in the data and affecting the precision of the findings.

Although the New York Heart Association (NYHA) functional classification and B-type natriuretic peptide (BNP) are valuable in assessing heart failure, they were excluded due to inconsistent documentation and evolving practice patterns. NYHA class was often subjectively recorded, and BNP levels were not routinely obtained prior to the 2019 AHA guidelines; even now, BNP measurement is recommended but not universally performed [1].

### Future Direction

In the future, a more controlled study may help to remove or significantly reduce confounding factors that can affect heart rate readings. Activity levels can vary greatly from patient to patient, significantly influencing readings like average and maximum heart rates. Additionally, 24-hour Holter monitors are limited in their utility due to the short period of time they capture. For instance, a child’s activity level may vary significantly between a school day, a weekend, and a day with sports participation. This variation may have impacted the overall data, and further studies are necessary to confirm these findings.

Single ventricle heart disease is found in 5 of 100,000 live births, and this study was partially limited by the number of available patients that fit our inclusion criteria [12]. Given the rarity of this physiology, performing a multi-center analysis is recommended to draw more definitive conclusions regarding heart rate variability in this unique set of patients.

## Conclusion

Patients with Fontan palliation are at high risk for sinus node dysfunction and arrhythmia [1, 6]. Resting ECG studies have suggested that these patients have lower resting heart rates [6]. This is the first study to describe heart rates in Fontan palliation via Holter monitor, providing a reference data set for routine screening. When compared to the single publication describing normal Holter values in healthy children, our Fontan population did not exhibit significantly lower heart rate minimums. Fontan patients under 4–6, 6–8, and 10–12 year old had significantly lower heart rate averages, though this may be skewed by lower heart rate maximums across the population. Within the Fontan cohort, minimum heart rates were significantly lower in male patients and those taking digoxin, while average heart rates were significantly lower in male patients and those taking beta-blockers only as well as those taking beta-blockers and digoxin. Additionally, patients with comorbid heterotaxy demonstrated higher heart rate minimums and averages. This is the first study to provide normative heart rate data for Fontan patients undergoing routine Holter monitor screening. Additional larger studies are necessary to confirm these findings and to provide better reference values for specific congenital heart defects and surgical palliations.

## Data Availability

Relevant data is provided within the manuscript. Supplementary or additional data available upon request.
